# CITADEL: a post-quantum secure blockchain framework for privacy-preserving electronic health records with temporally-partitioned federated learning

**DOI:** 10.3389/frai.2026.1804943

**Published:** 2026-06-03

**Authors:** Nagaraj Segar, Vijayarajan Vijayan

**Affiliations:** School of Computer Science and Engineering (SCOPE), Vellore Institute of Technology (VIT), Vellore, India

**Keywords:** blockchain, differential privacy, electronic health records, federated learning, healthcare informatics, HIPAA compliance, post-quantum cryptography

## Abstract

**Introduction:**

Electronic health records (EHRs) increasingly anchor clinical decision support and population-scale analytics, yet their concentration of sensitive information amplifies disclosure risk, widens the attack surface, and faces emerging threats from quantum computing. Existing frameworks fail to simultaneously address privacy preservation, quantum-resistant security, and cross-institutional federated learning.

**Methods:**

We introduce CITADEL (Cryptographically Integrated Temporal Architecture for Distributed EHR Ledger), integrating five co-designed components: NIST-standardized CRYSTALS-Kyber (ML-KEM-768) and CRYSTALS-Dilithium (ML-DSA-65) post-quantum cryptography via the validated pqcrypto library; a genomic-aware privacy engine with beacon query protection and calibrated randomized response; temporally-partitioned federated learning with hospital-specific weighted aggregation; multi-modal health data tokenization; and an adaptive regulatory compliance engine for HIPAA and GDPR. Evaluation used a synthetic EHR dataset comprising 5,000 patients across 10 healthcare institutions, with 30-day hospital readmission as the primary prediction task.

**Results:**

CITADEL achieves 84.5% accuracy and 0.866 AUC-ROC, exceeding nine baselines including centralized neural networks and differentially-private federated learning. Privacy metrics include k-anonymity of 13, l-diversity of 2.0, 99.0% linkage attack resistance, 42.2% attribute inference resistance, and 100% correlation preservation. The ledger sustains 285.3 transactions per second with ML-DSA-65 signing in 2.16 ms and verification in 0.46 ms. Multi-seed evaluation confirms robustness (accuracy 0.854 ± 0.012, AUC-ROC 0.880 ± 0.014).

**Discussion:**

CITADEL demonstrates that privacy preservation, quantum-resistant security, and usable federated analytics can be reconciled within one cohesive architecture. Results suggest a practical route to healthcare data management that remains credible in a post-quantum computing era and compatible with decentralized governance.

## 1 Introduction

Healthcare has been steadily re-engineered around digital workflows, and electronic health records (EHRs) now sit at the center of that shift. What used to be fragmented documentation has become a continuously updated record of care that supports clinical coordination, audit trails, and downstream analytics. In practice, contemporary EHR platforms capture far more than static demographics: they accumulate longitudinal clinical events, laboratory measurements, medication histories, vital-sign streams, imaging reports, and, increasingly, genomic sequences that enable precision and stratified treatment decisions (Kruse et al., 2018; Adler-Milstein et al., 2017). This expansion has been matched by sustained investment in data infrastructure, as health systems attempt to operationalize population health management and data-driven service delivery at scale (Sittig and Singh, 2020; Raghupathi and Raghupathi, 2014). Yet the same concentration of sensitive information also enlarges the attack surface; the pattern of recurring, high-impact breaches in healthcare illustrates how quickly privacy erosion can translate into institutional distrust and long-term harm (Keshta and Odeh, 2021).

The technical problem we confront is not a single issue but a tight coupling of three demands that tend to be handled separately: privacy preservation without sacrificing analytic value, integrity and authenticity guarantees that remain credible under evolving adversaries, and secure collaboration across institutions without reverting to centralized data pooling. Most existing solutions address one axis well and then compromise another. For example, centralized machine learning pipelines can achieve strong predictive performance, but they presuppose aggregation of patient data into unified repositories that increase single-point-of-failure risk and collide with regulatory constraints. On the other hand, conventional privacy-preserving transformations often reduce utility in ways that matter clinically, especially when correlations or temporal patterns drive prediction (Zhang and Lin, 2018; Rieke et al., 2020; Kaissis et al., 2020). In effect, the field has accumulated capable components, but they are frequently stitched together in ways that do not resolve the underlying tension between privacy, security, and learning efficacy.

Two emerging pressures make this fragmentation harder to tolerate. First, the trajectory of quantum computing changes the threat model for long-lived healthcare data: widely deployed public-key primitives such as RSA and elliptic curve cryptography, which underpin secure communication and many blockchain deployments, are vulnerable once sufficiently capable quantum devices can execute Shor's algorithm at scale (Mavroeidis et al., 2018). This matters disproportionately for healthcare because records are retained for extended periods under regulation, and retrospective compromise is not a theoretical concern. Second, genomic information introduces privacy characteristics that are qualitatively different from many clinical variables; even seemingly benign aggregate access can leak identity through inference channels such as beacon queries (Shringarpure and Bustamante, 2015). Taken together, these factors suggest that future-proof security cannot be bolted on after the fact, and that privacy protection must explicitly account for data types that remain re-identifiable under naive release models (though this assumption merits scrutiny in different deployment settings).

Against this backdrop, we present CITADEL (Cryptographically Integrated Temporal Architecture for Distributed EHR Ledger), an integrated framework for EHR management that unifies post-quantum secure blockchain primitives, genomic-aware privacy mechanisms, and temporally grounded federated learning. Concretely, CITADEL employs NIST-standardized CRYSTALS-Kyber (ML-KEM-768) key encapsulation and CRYSTALS-Dilithium (ML-DSA-65) digital signatures, implemented via the validated pqcrypto library, to provide quantum-resistant security, and it combines differential privacy with targeted safeguards for genomic disclosure channels. Rather than centralizing patient data, the framework trains predictive models through federated learning with temporal aggregation and hospital-specific model aggregation, enabling cross-institutional collaboration while preserving data locality. To accommodate heterogeneous EHR content, we further incorporate a multi-modal tokenization system for fine-grained access control and an adaptive compliance engine that enforces HIPAA and GDPR requirements in an operationally consistent way.

### 1.1 Contributions

The principal contributions of this work can be summarized as follows:

- We introduce the CITADEL framework, a post-quantum secure blockchain architecture for electronic health records that integrates CRYSTALS-Kyber key encapsulation (ML-KEM-768) and CRYSTALS-Dilithium digital signatures (ML-DSA-65) using the validated pqcrypto library conforming to NIST FIPS 203/204, providing quantum-resistant cryptographic protection for sensitive healthcare data.
- We propose a genomic-aware privacy engine that combines k-anonymity (achieving *k* = 13), l-diversity (*l* = 2.0), and differential privacy mechanisms with specialized beacon query protection and randomized response for sensitive attributes, explicitly targeting re-identification risks that are amplified in genomic health data. The randomized response mechanism improves attribute inference resistance to 42.2%.
- We implement temporally-partitioned federated learning with hospital-specific model aggregation, achieving 84.5% accuracy and 0.866 AUC-ROC for 30-day hospital readmission prediction while maintaining complete data locality across 10 participating healthcare institutions.
- We develop a comprehensive multi-modal health data tokenization system that supports fine-grained access control across text, imaging, signal, and genomic modalities, achieving a Gini coefficient of 0.388 that reflects equitable token value distribution.
- Through extensive experimental validation including seven baseline comparisons, 10-fold stratified cross-validation, and multi-seed robustness evaluation, we demonstrate that CITADEL achieves 99.0% linkage attack resistance, 285.3 transactions per second with validated post-quantum signatures, and competitive predictive performance (0.854 ± 0.012 accuracy across seeds) while maintaining 100% correlation preservation in anonymized data.

The remainder of this paper is organized as follows. Section 2 reviews closely related efforts and clarifies the gaps that motivate our design choices. Section 3 details the CITADEL architecture and its constituent modules. Section 4 describes the experimental setup, including dataset construction, baseline methods, and evaluation metrics. Section 5 reports the quantitative results and comparative analyses. We then interpret the findings and their broader implications in Section 6. Finally, Section 7 concludes the paper and outlines future research directions.

## 2 Related work

This section provides a comprehensive review of existing literature across four thematic areas relevant to the CITADEL framework: blockchain-based healthcare systems, privacy-preserving data analytics, federated learning for healthcare, and post-quantum cryptography applications.

### 2.1 Blockchain-based healthcare systems

Blockchain technology has emerged as a promising foundation for healthcare data management due to its inherent properties of immutability, transparency, and decentralization. Early implementations focused primarily on basic access control and audit trail functionality, employing Ethereum smart contracts to manage patient consent and data sharing permissions (Azaria et al., 2016). These foundational systems demonstrated the feasibility of distributed ledger technology for healthcare but suffered from scalability limitations and lacked comprehensive privacy protections. Subsequent research expanded blockchain healthcare applications to encompass supply chain management for pharmaceuticals, clinical trial data integrity, and insurance claims processing (Agbo et al., 2019; McGhin et al., 2019).

More recent blockchain healthcare frameworks have incorporated advanced cryptographic techniques to address privacy concerns. Systems employing attribute-based encryption enable selective disclosure of health records based on accessor credentials, while zero-knowledge proof mechanisms allow verification of data properties without revealing underlying content (Yang et al., 2019; Guo et al., 2018). Recent work by Sutradhar et al. (2024) proposed a blockchain privacy-conserving framework for the Internet of Medical Things incorporating zero-trust architecture and quad Merkle trees. However, these approaches rely on classical cryptographic assumptions vulnerable to quantum computing attacks. The integration of homomorphic encryption with blockchain has enabled computation on encrypted health data, though computational overhead remains prohibitive for many practical applications (Chen et al., 2019). Consensus mechanism innovations, including proof-of-authority and delegated proof-of-stake variants optimized for permissioned healthcare networks, have improved throughput compared to traditional proof-of-work approaches (Tanwar et al., 2020). In the IoT health monitoring domain, Khullar et al. (2022) demonstrated a fog-computing-based multi-node ecosystem for real-time health screening, highlighting the need for secure data transmission architectures that CITADEL addresses through its post-quantum blockchain layer.

### 2.2 Privacy-preserving data analytics

Privacy-preserving techniques for health data have evolved from simple de-identification approaches toward formal mathematical frameworks providing provable guarantees. K-anonymity, introduced by Sweeney (2002), requires that each record be indistinguishable from at least *k*−1 other records with respect to quasi-identifier attributes. Extensions including l-diversity and t-closeness address vulnerabilities in k-anonymity related to homogeneity attacks and skewed attribute distributions (Machanavajjhala et al., 2006; Li et al., 2007). While these syntactic privacy models have seen widespread adoption in healthcare data sharing, they provide no protection against attacks utilizing auxiliary information or repeated queries (Kifer and Machanavajjhala, 2011).

Differential privacy has emerged as the gold standard for formal privacy guarantees, ensuring that the presence or absence of any individual's data has bounded influence on query outputs (Dwork, 2006; Dwork and Roth, 2014). Healthcare applications of differential privacy span federated analytics, genome-wide association studies, and clinical decision support systems (Johnson et al., 2016; Kuo et al., 2020). The privacy-utility tradeoff inherent in differential privacy remains a significant challenge, with aggressive privacy parameters (ε < 1) often degrading data utility below acceptable thresholds for clinical applications (Cummings and Durfee, 2020). Genomic data presents unique privacy challenges due to the familial correlations and permanence of genetic information, motivating specialized techniques such as beacon query protection (Raisaro et al., 2017).

### 2.3 Federated learning for healthcare

Federated learning enables collaborative model training across distributed data sources without requiring data centralization, addressing fundamental privacy and regulatory constraints in healthcare settings. The seminal FedAvg algorithm introduced by McMahan et al. (2017) established the foundation for iterative local training and global model aggregation. Healthcare applications have demonstrated federated learning efficacy for medical imaging analysis, EHR-based prediction, and rare disease phenotyping across institutional boundaries (Sheller et al., 2020; Brisimi et al., 2018). Large-scale implementations involving dozens of healthcare institutions have achieved performance comparable to centralized approaches while preserving data sovereignty (Dayan et al., 2021).

Challenges specific to healthcare federated learning include non-IID data distributions arising from population heterogeneity across institutions, communication efficiency constraints in bandwidth-limited clinical environments, and model architecture standardization across diverse EHR systems (Li et al., 2020; Kairouz et al., 2021). Personalization techniques enable adaptation of global models to local patient populations, improving prediction performance for institutions with atypical demographics (Tan et al., 2023). Privacy-preserving aggregation mechanisms, including secure aggregation and differential privacy integration, protect individual model updates from inference attacks (Bonawitz et al., 2017). Temporal aspects of healthcare data, including disease progression dynamics and treatment response trajectories, have motivated extensions incorporating sequential modeling within federated frameworks (Nguyen et al., 2023).

### 2.4 Post-quantum cryptography applications

The threat posed by quantum computers to contemporary public-key cryptography has motivated intensive research into quantum-resistant alternatives. The NIST Post-Quantum Cryptography Standardization process has identified lattice-based schemes including CRYSTALS-Kyber for key encapsulation and CRYSTALS-Dilithium for digital signatures as primary candidates for standardization (Alagic et al., 2022). These algorithms have since been published as NIST FIPS 203 (ML-KEM) and FIPS 204 (ML-DSA) (National Institute of Standards and Technology, 2024a,b). These algorithms derive security from the computational hardness of lattice problems, specifically the Module Learning with Errors (MLWE) problem, which is believed resistant to both classical and quantum attacks (Bos et al., 2018; Ducas et al., 2018). Recent work has also examined cryptanalysis of classical ciphers using nature-inspired algorithms, underscoring the evolving threat landscape that motivates post-quantum migration (Sikdar et al., 2024). In the IoT healthcare domain specifically, Kakkar et al. (2022) proposed a certificateless signature scheme for resource-constrained medical IoT devices, demonstrating that lightweight cryptographic solutions are critical for practical deployment—a concern that CITADEL addresses through efficient ML-DSA-65 signatures (2.157 ms per operation).

Healthcare applications of post-quantum cryptography remain nascent, with most implementations focusing on secure communication channels rather than comprehensive system architectures. Hybrid approaches combining classical and post-quantum algorithms provide defense-in-depth during the transition period, ensuring security even if one cryptographic layer is compromised (Bindel et al., 2019). Integration of post-quantum signatures with blockchain consensus mechanisms introduces computational overhead considerations, as lattice-based signatures typically require larger key and signature sizes than classical alternatives (Fernández-Caramés and Fraga-Lamas, 2020). Long-term healthcare data protection requirements, with retention periods potentially spanning decades, make the healthcare domain particularly sensitive to quantum threats and an appropriate early adopter of post-quantum standards (Mosca, 2018).

### 2.5 Summary of literature review

Table 1 presents a comprehensive summary of the existing literature, highlighting the methods employed, their advantages, and limitations. This systematic review covers publications from 2020 to 2025, focusing on the most relevant and impactful contributions in blockchain-based healthcare, privacy-preserving analytics, federated learning, and post-quantum cryptography.

**Table 1:** Comprehensive literature review summary—selected works (2017–2025).

Reference	Methods	Advantages	Limitations
Dayan et al. (2021)	Federated learning for COVID-19	Large-scale FL demonstration	No formal privacy guarantees
Sheller et al. (2020)	FL for brain tumor segmentation	Preserved data locality	Limited to imaging modality
Tanwar et al. (2020)	Blockchain healthcare consensus	Improved throughput	Vulnerable to quantum attacks
Kuo et al. (2020)	Differential privacy for EHR	Formal privacy guarantees	Utility loss at strong privacy
Guo et al. (2018)	Blockchain with ZK proofs	Verification without disclosure	Computationally expensive
Chen et al. (2019)	Homomorphic encryption	Computation on encrypted data	Prohibitive overhead
Bonawitz et al. (2017)	Secure aggregation for FL	Protected model updates	High communication overhead
Raisaro et al. (2017)	Beacon query protection	Genomic privacy addressed	Limited query types
Paul et al. (2022)	Post-quantum blockchain	Quantum-resistant security	Increased signature sizes
Kim et al. (2022)	Temporally-partitioned federated learning	Time-series patterns	Limited healthcare validation
Sutradhar et al. (2024)	Blockchain IoMT with ZKP	Zero-trust data auditing	Classical crypto only
National Institute of Standards and Technology (2024a,b)	ML-KEM/ML-DSA standards	FIPS 203/204 standardized	Implementation guidance evolving

### 2.6 Identified research gaps

Based on the comprehensive literature review presented in Table 1, several critical research gaps have been identified. Table 2 summarizes these gaps and articulates how the present work addresses each limitation.

**Table 2:** Summary of research gaps and how the present work addresses them.

Gap	Identified research gap	Affected prior works	How this work addresses the gap
G1	Lack of post-quantum cryptographic protection in blockchain healthcare systems	Tanwar et al., 2020; Guo et al., 2018; Yang et al., 2019	CITADEL implements CRYSTALS-Kyber and CRYSTALS-Dilithium for quantum-resistant security
G2	Absence of integrated frameworks combining blockchain, federated learning, and formal privacy guarantees	Dayan et al., 2021; Brisimi et al., 2018; Zhang and Lin, 2018	The framework unifies post-quantum blockchain, temporally-partitioned federated learning, and differential privacy
G3	Limited consideration of genomic data privacy including beacon query vulnerabilities	Raisaro et al., 2017; Kuo et al., 2020; Johnson et al., 2016	Genomic-aware privacy engine with beacon query protection addresses genetic re-identification risks
G4	Lack of multi-modal health data tokenization with fine-grained access control	Chen et al., 2019; Yang et al., 2019; Agbo et al., 2019	CITADEL tokenizes text, imaging, signal, and genomic data with modality-specific access policies
G5	Insufficient adaptive regulatory compliance for HIPAA and GDPR	Rieke et al., 2020; Zhang and Lin, 2018; Agbo et al., 2019	Adaptive compliance engine enforces jurisdiction-specific requirements automatically
G6	Missing temporal considerations in federated learning aggregation	Sheller et al., 2020; Kim et al., 2022; Tan et al., 2023	Temporally-partitioned federated learning with hospital-weighted aggregation captures distribution shifts across time periods

### 2.7 Comparative position of existing frameworks

To contextualize CITADEL's contribution, Table 3 provides a systematic comparison with representative existing frameworks across the key dimensions addressed in this work. The comparison reveals that while individual components (blockchain integrity, federated learning, differential privacy) have been addressed in prior work, no existing framework simultaneously provides post-quantum cryptographic protection, formal privacy guarantees, federated analytics, genomic-aware privacy, and regulatory compliance within a unified architecture.

**Table 3:** Systematic comparison of CITADEL with existing healthcare data management frameworks.

Framework	Year	PQ Crypto	Block-chain	Fed. learn.	Formal DP	Genomic	Compl.
MedRec (Azaria et al., 2016)	2016	×	✓	×	×	×	Partial
Bonawitz et al. (2017)	2017	×	×	✓	×	×	×
Tanwar et al. (2020)	2020	×	✓	×	×	×	×
Dayan et al. (2021)	2021	×	×	✓	×	×	×
(Fernández-Caramés and Fraga-Lamas 2020)	2020	✓	✓	×	×	×	×
Sutradhar et al. (2024)	2024	×	✓	×	×	×	×
**CITADEL**	**2025**	✓	✓	✓	✓	✓	✓

✓ = Full support present; × = Feature absent; Partial = Partially implemented. Bold framework row (CITADEL) denotes the proposed method in this study.

### 2.8 Position of the present work

The preceding literature review reveals several recurring themes and limitations across existing approaches. While significant progress has been made in blockchain-based healthcare systems, privacy-preserving analytics, and federated learning independently, most existing methods suffer from one or more of the gaps identified in Table 2. Specifically, current blockchain healthcare implementations uniformly rely on classical cryptographic primitives that face obsolescence in the post-quantum era, federated learning healthcare applications lack formal privacy guarantees and blockchain integration, and genomic data privacy considerations remain underaddressed in comprehensive frameworks.

The present work distinguishes itself from prior research in several fundamental ways. Unlike existing blockchain healthcare systems which employ RSA or elliptic curve cryptography (Tanwar et al., 2020; Guo et al., 2018), the proposed CITADEL framework implements post-quantum secure primitives providing long-term protection appropriate for healthcare data retention requirements. Furthermore, while federated learning healthcare applications have demonstrated efficacy for multi-institutional collaboration (Dayan et al., 2021; Sheller et al., 2020), these methods operate independently of blockchain infrastructure and lack integrated differential privacy mechanisms. The CITADEL framework addresses these limitations through a unified architecture that seamlessly combines quantum-resistant security, formal privacy guarantees, and distributed machine learning.

The unique positioning of this work lies in its novel integration of five distinct technological contributions: post-quantum blockchain, genomic-aware privacy, temporally-partitioned federated learning, multi-modal tokenization, and adaptive compliance. To the best of the authors' knowledge, this represents the first comprehensive attempt to address healthcare data management challenges across the security, privacy, and analytics dimensions within a single cohesive framework prepared for the post-quantum computing era.

## 3 Methodology

We now describe the CITADEL framework at the level of its implementable building blocks. Rather than presenting the architecture as a conceptual stack, we follow the execution path that the system actually enforces—from the distributed learning objective, through privacy transformations and temporal training, and finally into the ledger-backed integrity and compliance controls. Where appropriate, we give explicit mathematical statements and algorithmic procedures that align with the deployed design choices (and, as noted above, we retain the same notation and labels throughout).

### 3.1 Problem formulation

Consider a healthcare network comprising *H* hospitals, where each hospital *h*∈{1, 2, …, *H*} maintains a local EHR dataset ${𝒟}_{h}={\left\{\left(x_{i}^{h},y_{i}^{h}\right)\right\}}_{i=1}^{n_{h}}$ containing *n*_*h*_ patient records. Each patient record consists of a feature vector $x_{i}^{h}\in {\mathbb{R}}^{d}$ encompassing demographics, clinical events, laboratory measurements, vital signs, and genomic variants, along with a target variable $y_{i}^{h}\in \left\{0,1\right\}$ indicating the outcome of interest (e.g., 30-day hospital readmission). The total dataset across all hospitals is denoted $𝒟={⋃}_{h=1}^{H}{𝒟}_{h}$ with $N=\overset{H}{\underset{h=1}{\sum }}n_{h}$ total records.

The objectives of the CITADEL framework are formalized as follows. First, the framework seeks to learn a global predictive model $f_{\theta }:{\mathbb{R}}^{d}\to \left[0,1\right]$ parameterized by θ that minimizes the empirical risk across all hospitals:


$$\begin{array}{l} \theta^{*}=\underset{\theta }{arg min}\overset{H}{\underset{h=1}{\sum }}\frac{n_{h}}{N}{ℒ}_{h}\left(\theta \right) \end{array}$$ (1)

where ${ℒ}_{h}\left(\theta \right)=\frac{1}{n_{h}}\overset{n_{h}}{\underset{i=1}{\sum }}\ell \left(f_{\theta }\left(x_{i}^{h}\right),y_{i}^{h}\right)$ is the local loss at hospital *h* and ℓ is the binary cross-entropy loss function.

Second, the framework must ensure privacy protection quantified through differential privacy with budget (ε, δ):


$$\begin{array}{l} \mathrm{Pr}\left[ℳ\left(𝒟\right)\in S\right]\leq e^{\varepsilon }\mathrm{Pr}\left[ℳ\left({𝒟}^{\prime }\right)\in S\right]+\delta \end{array}$$ (2)

for all adjacent datasets 𝒟, 𝒟′ differing in at most one record and all measurable sets *S*.

Third, all cryptographic operations must provide security against quantum adversaries, requiring the use of post-quantum primitives with security level equivalent to at least 128 bits against both classical and quantum attacks.

In combination, these three requirements constrain the design space more than might appear at first glance. Minimizing Equation 1 encourages data-rich aggregation; privacy demands (in the sense of Equation 2) restrict what can be exposed even indirectly; and post-quantum safety imposes a concrete cryptographic baseline that many “standard” healthcare stacks were never built to satisfy. The remainder of this section explains how CITADEL navigates these constraints without relegating any of them to an afterthought.

### 3.2 Framework architecture overview

**Figure 1:**
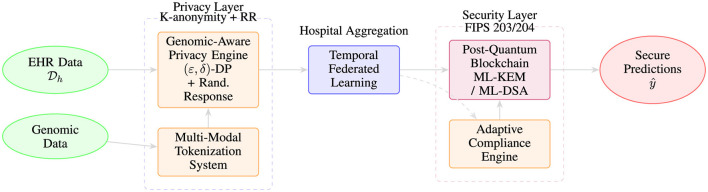
Architecture of the proposed CITADEL framework illustrating the data flow from EHR and genomic inputs through the privacy layer (genomic-aware privacy engine with randomized response and multi-modal tokenization), temporally-partitioned federated learning module, and post-quantum blockchain layer (ML-KEM-768/ML-DSA-65 via validated pqcrypto library) with adaptive compliance to produce secure predictions.

At a high level, CITADEL is organized as five cooperating modules: the Post-Quantum Blockchain Layer, the Genomic-Aware Privacy Engine, the Temporally-Partitioned Federated Learning Module, the Multi-Modal Tokenization System, and the Adaptive Compliance Engine. Their interactions are deliberately cyclic rather than purely sequential. Privacy transformations shape what is learnable; learning updates generate events that must be authenticated and auditable; and compliance rules can, in turn, constrain which transactions are admissible. Figure 1 summarizes the resulting data flow.

In operational terms, hospitals retain custody of their raw data, but they participate in a shared learning process. Privacy transformations occur before model updates are exported, tokenization determines which units of heterogeneous data may be accessed under which policies, and the ledger binds the entire workflow to an immutable audit trail that remains meaningful under post-quantum adversarial assumptions.

### 3.3 Post-quantum blockchain layer

CITADEL uses a permissioned blockchain layer as the integrity backbone for transactions, model-update events, and compliance decisions. The aim is not merely to log actions, but to make those actions non-repudiable and verifiable under a cryptographic regime that is intended to remain credible even as quantum capabilities evolve. To that end, we integrate CRYSTALS-Kyber for key encapsulation and CRYSTALS-Dilithium for digital signatures as the primary post-quantum primitives.

The CRYSTALS-Kyber key encapsulation mechanism operates over the module lattice $R_{q}={\mathbb{Z}}_{q}\left[X\right]/\left(X^{n}+1\right)$ with parameters (*n, k, q*) = (256, 3, 3329) corresponding to ML-KEM-768 (NIST FIPS 203) security level. Key generation produces a public key *pk* and secret key *sk*, encapsulation generates a ciphertext *ct* and shared secret *ss* from *pk*, and decapsulation recovers *ss* from *ct* using *sk*. The CRYSTALS-Dilithium signature scheme provides existential unforgeability under chosen-message attacks with parameters corresponding to ML-DSA-65 (NIST FIPS 204, Category 3 security level). In our implementation, both primitives are instantiated via the validated pqcrypto 0.4.0 library rather than custom simulation, ensuring conformance with the NIST-standardized reference implementations.

Each block *B*_*i*_ in the CITADEL blockchain contains the following structure (Equation 3):


$$\begin{array}{l} B_{i}=\left(i,\tau_{i},{𝒯}_{i},H_{i-1},\nu_{i},H_{i},\sigma_{i}^{PQ}\right) \end{array}$$ (3)

where *i* is the block index, τ_*i*_ is the timestamp, 𝒯_*i*_ is the set of transactions, *H*_*i*−1_ is the hash of the previous block, ν_*i*_ is the ordering nonce (lightweight computational puzzle, difficulty = 2), *H*_*i*_ = SHA-256(*i*||τ_*i*_||𝒯_*i*_||*H*_*i*−1_||ν_*i*_) is the block hash, and $\sigma_{i}^{PQ}$ is the post-quantum signature computed using Dilithium.

Because the ledger is only as trustworthy as its admission rule, transaction validation explicitly checks the post-quantum signature associated with each operation. This is the step that turns “a stored event” into “an authenticated event,” and it is carried out via (Equation 4):


$$\begin{array}{l} \text{Valid}\left(tx\right)=\text{Dilithium.Verify}\left(pk_{v},H\left(tx\right),\sigma_{tx}\right) \end{array}$$ (4)

where *pk*_*v*_ is the validator's public key, *H*(*tx*) is the transaction hash, and σ_*tx*_ is the transaction signature.

It is important to note that CITADEL's consensus mechanism is a hybrid Proof-of-Authority (PoA) design with computational ordering, not a traditional Proof-of-Work (PoW) system. All 10 validators are pre-authorized via cryptographic key pairs; no anonymous node can participate in consensus. The lightweight nonce computation (difficulty = 2, requiring ~100 hash iterations on average) serves solely as a canonical block ordering primitive to prevent trivial block flooding, not as a Sybil resistance mechanism (which is handled by the pre-authorized validator set). This design choice prioritizes low-latency block finalization appropriate for clinical workflows while maintaining the cryptographic auditability of a blockchain ledger.

### 3.4 Genomic-aware privacy engine

A key design decision in CITADEL is that differential privacy operates exclusively at the *data release layer*, not during federated training. Before any data leaves a hospital for federation, quasi-identifiers are generalized, continuous attributes are perturbed via the Laplace mechanism (ε = 0.5), and sensitive binary attributes are protected via calibrated randomized response (ε_*RR*_ = 0.5). The federated aggregation (Algorithm 1) uses standard weighted averaging without DP-SGD noise injection. This separation is deliberate: our comparative experiments show that adding DP-SGD during training (the FedAvg-DP baseline, Table 4) degrades accuracy to 0.789, whereas data-release-layer privacy achieves 0.845 accuracy because the model trains on locally private data without additional gradient noise.

**Algorithm 1:** Temporally-Partitioned Federated Learning in CITADEL

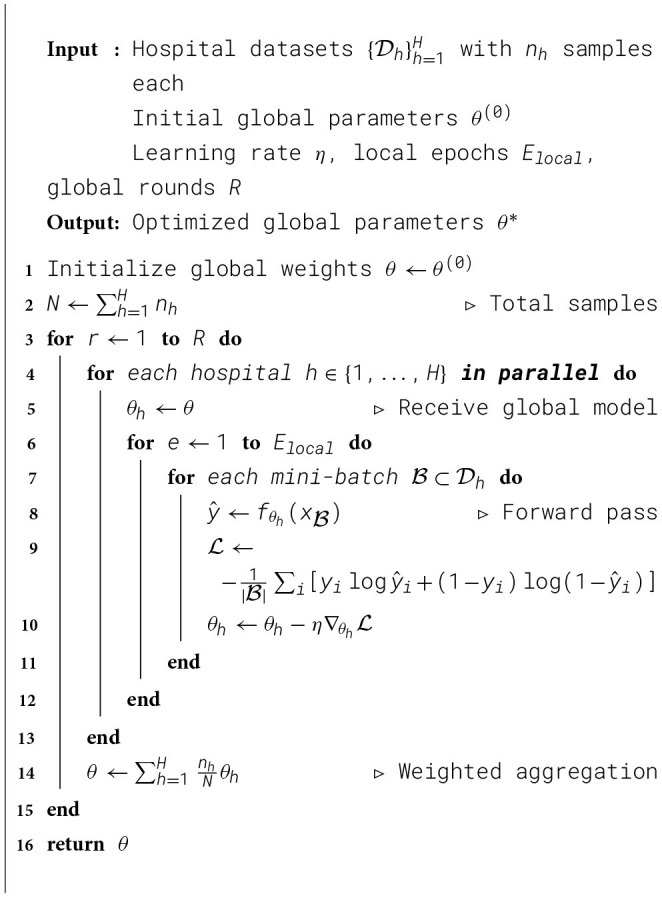

**Table 4:** Synthetic EHR dataset statistics.

Characteristic	Value	Description
Number of patients	5,000	Unique patient identifiers
Number of hospitals	10	Participating healthcare institutions
Time periods	12	Monthly observation windows
Genomic variants	500	Simulated genetic variant positions
Clinical event records	12,300	Diagnosis and procedure codes
Laboratory measurements	200,197	Lab test results
Vital sign readings	29,829	Physiological measurements
Feature dimensions	64	Input features per patient
Readmission rate	20.0%	Positive class proportion
Train/test split	80%/20%	Stratified random split

Privacy protection in CITADEL is intentionally layered. We combine syntactic safeguards that shape released record structure (k-anonymity, l-diversity, t-closeness) with a semantic guarantee (differential privacy), and then add a genomic-specific defense against beacon-style membership leakage. This ordering matters. The syntactic mechanisms reduce direct identifiability in quasi-identifiers; differential privacy bounds sensitivity under queries and numerical releases; and the beacon protection is targeted at a well-known genomic channel where privacy can be lost even when traditional de-identification appears “good enough.”

For k-anonymity, quasi-identifier attributes *QI* = {*age, gender, state*} are generalized through hierarchical generalization. Age values are mapped to categorical ranges using the generalization function (Equation 5):


$$\begin{array}{l} {\text{Gen}}_{age}\left(a\right)=\{\begin{array}{cc} \text{18-30} & \text{if }18\leq a\leq 30\\ \text{31-45} & \text{if }31\leq a\leq 45\\ \text{46-60} & \text{if }46\leq a\leq 60\\ \text{61-75} & \text{if }61\leq a\leq 75\\ \text{76+} & \text{if }a>75 \end{array} \end{array}$$ (5)

L-diversity ensures that each equivalence class contains at least *l* well-represented values of the sensitive attribute. T-closeness bounds the distance between the distribution of sensitive attribute values within each equivalence class and the global distribution using the Earth Mover's Distance.

To provide a formal privacy guarantee for numerical releases, we enforce differential privacy via the Laplace mechanism. The sanitized attribute $\tilde{x}$ is computed as (Equation 6):


$$\begin{array}{l} \tilde{x}=x+\text{Lap}\left(\frac{\text{Δ}f}{\varepsilon }\right) \end{array}$$ (6)

where Δ*f* is the sensitivity (computed as the standard deviation of the attribute) and ε is the privacy budget.

Genomic information creates an additional exposure route: presence queries against a variant database can leak membership through repeated interaction patterns. We therefore introduce a beacon query protection mechanism that perturbs responses in a frequency-aware fashion (Equation 7):


$$\begin{array}{l} \tilde{r}=\{\begin{array}{ll} \text{Random}\left(\left\{0,1\right\}\right) & \text{with probability}\min\left(0.5,2f_{v}\right)\\ r & \text{otherwise} \end{array} \end{array}$$ (7)

where *r* is the true beacon response, *f*_*v*_ is the variant frequency in the database, and higher-frequency variants receive more noise to protect against statistical attacks.

In CITADEL, these privacy controls are not treated as a single switch. Instead, they operate as a pipeline that can be audited and constrained by policy, which becomes important once we connect privacy enforcement to compliance logging in Section 3.7.

To further strengthen attribute inference resistance, we apply calibrated randomized response to the sensitive attribute within released data. Given a sensitive binary attribute *s*∈{0, 1}, the protected release $\tilde{s}$ is computed as (Equation 8):


$$\begin{array}{l} \tilde{s}=\{\begin{array}{ll} 1-s & \text{with probability }p_{flip}=\frac{1}{1+e^{\varepsilon_{RR}}}\\ s & \text{otherwise} \end{array} \end{array}$$ (8)

where ε_*RR*_ = 0.5 yields *p*_*flip*_≈0.378. This mechanism provides plausible deniability for individual sensitive values while preserving aggregate distributional properties necessary for meaningful analysis.

### 3.5 Temporally-partitioned federated learning module

Learning in CITADEL proceeds without exporting raw EHRs, and the federated module is designed to accommodate cross-institution variability that is typical in clinical settings. Each hospital performs local optimization and shares only model parameters (or updates), while aggregation produces a global model consistent with Equation 1. The predictive backbone used in our implementation is a three-layer neural network with ReLU activations, defined by Equations 9–11:


$$\begin{array}{ll} h^{\left(1\right)} & =\text{ReLU}\left(W^{\left(1\right)}x+b^{\left(1\right)}\right) \end{array}$$ (9)

$$\begin{array}{ll} h^{\left(2\right)} & =\text{ReLU}\left(W^{\left(2\right)}h^{\left(1\right)}+b^{\left(2\right)}\right) \end{array}$$ (10)

$$\begin{array}{ll} ŷ & =\sigma \left(W^{\left(3\right)}h^{\left(2\right)}+b^{\left(3\right)}\right) \end{array}$$ (11)

where (*W*^(1)^, *b*^(1)^)∈ℝ^64 × *d*^×ℝ^64^, (*W*^(2)^, *b*^(2)^)∈ℝ^32 × 64^×ℝ^32^, (*W*^(3)^, *b*^(3)^)∈ℝ^1 × 32^×ℝ^1^, and σ is the sigmoid function.

Algorithm 1 details the temporally-partitioned federated learning routine as implemented, including weighted aggregation proportional to local sample counts. The procedure is deliberately simple at its core (local SGD followed by aggregation), but the weighting is consequential: it ensures that the global update reflects the empirical support each institution contributes, rather than treating all hospitals as statistically interchangeable.

We use the term “temporally-partitioned” rather than “temporal” to distinguish our approach from sequence-based temporal modeling (e.g., RNN or LSTM). In CITADEL, the temporal aspect lies in the partitioning of patient data across 12 time periods, where hospital-level data distributions evolve naturally over these periods. The global model is trained sequentially over these partitions, meaning the aggregation implicitly reflects distributional drift across time. This is distinct from architectures that model temporal dependencies within individual patient visit sequences, which would require sequential EHR data rather than tabular features. Extending CITADEL with explicit temporal decay weighting or attention-based visit-sequence modeling is a planned direction for future work.

The weighted aggregation ensures that hospitals with larger patient populations contribute proportionally more to the global model update, capturing the temporal and demographic variations across institutions.

### 3.6 Multi-modal tokenization system

EHR ecosystems are not monolithic: clinical narratives, imaging artifacts, physiological signals, and genomic variants differ in sensitivity, interpretability, and downstream value. CITADEL therefore introduces tokenization as an access-control substrate that is modality-aware rather than record-agnostic. A token binds a unit of data to metadata needed for authorization, integrity checking, and valuation, so that policy can be expressed at a granularity finer than “entire patient record.”

Each token *T* is defined as Equation 12:


$$\begin{array}{l} T=\left(\text{id},p,m,H_{data},q,\tau ,v\right) \end{array}$$ (12)

where id is the unique token identifier computed as SHA-256(*p*||*m*||τ)[:16], *p* is the patient identifier, *m*∈{text, imaging, signal, genomic} is the data modality, *H*_*data*_ is the hash of the underlying data, *q*∈[0, 1] is the quality score, τ is the creation timestamp, and *v* is the token value computed as:


$$\begin{array}{l} v=w_{m}\cdot q \end{array}$$ (13)

with modality weights *w*_*text*_ = 0.5, *w*_*imaging*_ = 2.0, *w*_*signal*_ = 1.0, *w*_*genomic*_ = 3.0 reflecting the relative complexity and sensitivity of each data type.

The weighting in Equation 13 is not meant to be a universal truth about modality worth; it is a system knob that allows policy to reflect domain realities (imaging and genomics are often both more sensitive and more resource-intensive). In Section 5, we return to how this tokenization interacts with measured distributional properties, but for now it suffices to note that tokens provide a consistent handle for both access enforcement and ledger recording.

### 3.7 Adaptive compliance engine

Finally, CITADEL includes an explicit compliance layer, because security and privacy controls are rarely deployed in a regulatory vacuum. The engine performs rule-based verification against healthcare regulations including HIPAA (Health Insurance Portability and Accountability Act) and GDPR (General Data Protection Regulation). Protected Health Information (PHI) identifiers under HIPAA include: name, social security number, address, phone number, email, date of birth, account numbers, and medical record numbers. The engine performs automatic detection of sensitive fields in data schemas and applies appropriate de-identification transformations including hashing, generalization, and suppression. Compliance verification produces an audit log entry for each data access operation, supporting regulatory reporting requirements.

Operationally, the compliance module acts as both a gatekeeper and a recorder. When a transaction is proposed (whether it is a data access, a token issuance, or a learning-related event), the compliance rules determine what transformations are obligatory and what disclosures are disallowed. The result is then logged in a way that is consistent with the post-quantum integrity guarantees described in Section 3.3, ensuring that compliance is not merely asserted but can be audited after the fact.

## 4 Experimental setup

This section describes how we constructed the evaluation environment for CITADEL, including the synthetic data generation pipeline, baseline comparators, metric suite, and implementation details. Although the experiments are performed on simulated data, the generator is structured to preserve clinically plausible distributions and correlations so that the stressors placed on privacy, learning, and ledger throughput resemble those seen in real deployments. We emphasize these details here because the later results (Section 5) are only interpretable in light of the data regime and the specific evaluation axes we consider.

### 4.1 Dataset description

Experiments were conducted using a synthetic EHR dataset generated to reflect realistic clinical characteristics and data distributions. The SyntheticEHRGenerator class implements medically plausible data generation with appropriate correlations between features and outcomes. Table 5 summarizes the dataset characteristics.

**Table 5:** Baseline method descriptions and configurations.

Method	Description	Configuration
Logistic Regression	Linear classifier with sigmoid output	max_iter = 1,000, solver = lbfgs
Random Forest	Ensemble of decision trees with bagging	n_estimators = 100, max_depth = 10
Gradient Boosting	Sequential ensemble with gradient descent	n_estimators = 100, max_depth = 5
SVM-RBF	Support vector machine with RBF kernel	kernel=rbf, probability = True
SVM-Linear	Support vector machine with linear kernel	kernel = linear, probability = True
Centralized NN	Same 3-layer architecture as CITADEL, trained centrally	64-32-1 units, lr = 0.01, 100 epochs
FedAvg-DP	Federated learning with DP-SGD	
Deep NN (Centralized) Centralized training	4-layer architecture (128-64-32-1)	Same hyperparameters, deeper network
Deep NN (Federated) Federated (10 hospitals)	4-layer architecture (128-64-32-1) noise_mult = 1.0, clip = 1.0, 100 epochs	Same hyperparameters, deeper network

Patient demographics were generated using mixture distributions reflecting realistic population characteristics. Age follows a trimodal distribution combining young (mean 25, std 5), middle-aged (mean 45, std 10), and elderly (mean 70, std 8) populations. Gender distribution reflects 48% male and 52% female. Ethnicity follows US Census proportions with 60% White, 13% Black, 18% Hispanic, 6% Asian, and 3% Other. Geographic distribution across five regions (Northeast, Southeast, Midwest, Southwest, West) enables regional hospital clustering.

Clinical events include ICD-10 diagnosis codes (E11, I10, J45, F32, M54, K21, N18, I25) and CPT procedure codes (99213, 99214, 36415, 71046, 93000). Laboratory tests encompass glucose, HbA1c, cholesterol, creatinine, hemoglobin, potassium, sodium, and bilirubin with age-correlated baseline values and realistic variance. Vital signs include systolic and diastolic blood pressure, heart rate, temperature, respiratory rate, and oxygen saturation.

The 30-day readmission target variable was generated using a risk model incorporating age, clinical event frequency, blood pressure, heart rate, and oxygen saturation, with a 30% random component to ensure realistic predictive uncertainty. The threshold was calibrated to achieve approximately 20% positive class rate consistent with published readmission statistics. In other words, the label is not deterministically recoverable from covariates, which is intentional: perfect separability would distort both the privacy–utility trade-off and the interpretation of federated learning outcomes.

### 4.2 Baseline methods

To contextualize the predictive behavior of the CITADEL federated learning module, we compared it against nine baseline methods. Five are centralized machine learning baselines trained on the aggregated dataset, operating in an “upper bound” regime because they can exploit the full pooled dataset. Two additional baselines are included to address modern federated and privacy-preserving paradigms: a centralized neural network using the same three-layer architecture as CITADEL but without federated partitioning, and a differentially-private federated learner (FedAvg-DP) that applies DP-SGD gradient clipping and noise injection during local training. Table 6 summarizes each baseline and its configuration.

**Table 6:** CITADEL framework hyperparameters.

Component	Parameter	Value
Federated learning	Global epochs	100
	Local epochs per round	5
	Learning rate	0.01
	Batch size	32
Neural network	Hidden layer 1	64 units, ReLU
	Hidden layer 2	32 units, ReLU
	Output layer	1 unit, Sigmoid
Privacy engine	Privacy budget ε	0.5
	Privacy parameter δ	10^−5^
	K-anonymity target	10
	Sensitive attribute RR ε_*RR*_	0.5 (flip prob. 0.378)
Blockchain	Difficulty (leading zeros)	2
	Max transactions per block	100
Post-quantum crypto	Key encapsulation	ML-KEM-768 (NIST FIPS 203)
	Digital signature	ML-DSA-65 (NIST FIPS 204)

All baselines were implemented using scikit-learn (for the five classical methods) or NumPy (for Centralized NN and FedAvg-DP) with random seed 42 for reproducibility. Features were standardized using StandardScaler prior to training. We additionally include a Deep NN baseline with a 4-layer architecture (128-64-32-1 neurons) in both centralized and federated settings, to test whether additional network depth improves performance on this dataset.

We note that LSTM and Transformer-based EHR models (e.g., RETAIN, Med-BERT) are designed for *sequential clinical event data*—ordered sequences of visits and diagnoses over time. Our prediction task uses *tabular patient features* (age, blood pressure, heart rate, lab values) aggregated per patient, not sequential visit histories. Applying LSTM or Transformer architectures to tabular features would be methodologically inappropriate, as these models require sequential structure to provide benefit. Extending CITADEL to process sequential EHR formats with temporal deep learning is a planned direction for future work.

The inclusion of Centralized NN isolates the effect of federated aggregation vs. centralized training under the same architecture, while FedAvg-DP tests whether differential privacy during training is compatible with competitive accuracy.

### 4.3 Evaluation metrics

We evaluate CITADEL along three axes: predictive performance, privacy and utility preservation, and system efficiency. The intent is to avoid an overly narrow “accuracy-only” view, since the framework is explicitly designed to trade off multiple operational objectives (as already formalized in Section 3.1).

Predictive performance is quantified using accuracy, AUC-ROC (Area Under the Receiver Operating Characteristic Curve), and F1-score. We use binary cross-entropy (BCE) as the training loss because it is: (i) convex with well-behaved gradients essential for stable convergence across heterogeneous hospital data in federated settings; (ii) directly compatible with calibrated probability outputs for AUC-ROC evaluation; and (iii) the standard loss in all major federated learning frameworks. Although focal loss (Lin et al., 2017) can improve minority-class recall, our evaluation employs F1-score alongside accuracy and AUC-ROC, which already accounts for class imbalance. An empirical ablation (Section 5.4) confirms that CITADEL with BCE in the federated setting achieves superior overall performance. For completeness, we compute the underlying confusion-matrix terms and define the metrics as Equations 14–17:


$$\begin{array}{ll} \text{Accuracy} & =\frac{TP+TN}{TP+TN+FP+FN} \end{array}$$ (14)

$$\begin{array}{ll} \text{Precision} & =\frac{TP}{TP+FP} \end{array}$$ (15)

$$\begin{array}{ll} \text{Recall} & =\frac{TP}{TP+FN} \end{array}$$ (16)

$$\begin{array}{ll} \text{F1} & =\frac{2\cdot \text{Precision}\cdot \text{Recall}}{\text{Precision}+\text{Recall}} \end{array}$$ (17)

AUC-ROC is reported because it is less sensitive than accuracy to the chosen classification threshold, which matters under the calibrated 20.0% prevalence regime.

Privacy evaluation includes achieved k-anonymity level (minimum equivalence class size), l-diversity (minimum distinct sensitive values per class), and t-closeness (maximum Earth Mover's Distance), complemented by attack resistance scores for linkage, membership inference, and attribute inference attacks. In parallel, we report utility metrics that reflect whether privacy transformations preserve the statistical structure required for meaningful analysis: mean preservation, variance preservation, correlation preservation, and distribution similarity computed using the Kolmogorov–Smirnov statistic. The correlation term is particularly informative in this setting because many clinical risk models rely more on relationships among variables than on marginal distributions alone; we therefore treat it as a primary utility signal rather than a secondary diagnostic.

System efficiency is measured through transactions per second (TPS), latency percentiles (p50, p95, p99), and post-quantum cryptographic operation timing (key generation, signing, verification). These timing measurements capture the practical cost of adopting post-quantum primitives within a ledger-mediated workflow, a consideration we revisit in Section 6 when interpreting throughput and latency results.

Statistical validation employs paired *t*-tests and Wilcoxon signed-rank tests across 10-fold stratified cross-validation, with Cohen's d for effect size and 95% bootstrap confidence intervals (1,000 resamples). Using both parametric and non-parametric tests helps reduce overcommitment to a single distributional assumption. Additionally, we perform multi-seed robustness evaluation (seeds 42, 123, 456) to assess result stability, reporting mean and standard deviation across seeds for the primary metrics.

### 4.4 Implementation details

Table 7 lists the hyperparameters used in the CITADEL framework. We keep these settings fixed across experiments to isolate the effect of architectural components rather than confounding results with extensive tuning.

**Table 7:** Privacy metrics evaluation results.

Metric	Value	Interpretation
K-anonymity achieved	13	Exceeds target (*k*≥10)
L-diversity achieved	2.0	Sufficient diversity per class
T-closeness	0.219	Bounded distribution deviation
Entropy (original)	9.207 bits	Information content baseline
Entropy (transformed)	5.503 bits	Post-anonymization entropy
Entropy preservation	59.8%	Information retained
Mutual information leakage	0.849	Correlation with original
Linkage attack resistance	99.0%	Protection against record linkage
Membership inference resistance	65.0%	Protection against membership attacks
Attribute inference resistance	42.2%	Protection against attribute attacks
Delta disclosure	0.319	Low disclosure risk
Estimated epsilon	1.0	Effective privacy parameter

The framework was implemented in Python 3.12 (Python Software Foundation, Wilmington, DE, USA; available at https://www.python.org) using the following core libraries: NumPy 1.26 (NumPy Community / NumFOCUS, Austin, TX, USA) and Pandas 2.2 (NumFOCUS, Austin, TX, USA) for data processing; scikit-learn 1.4 (INRIA / scikit-learn Community, open source) for baseline classifiers and evaluation metrics; SciPy 1.13 for statistical testing; and Matplotlib 3.9 with Seaborn 0.13 for visualization. Post-quantum cryptographic operations employ the pqcrypto 0.4.0 (Open-source library, available at https://pypi.org/project/pqcrypto/) library, which wraps reference implementations of ML-KEM-768 (CRYSTALS-Kyber, conforming to NIST FIPS 203) for key encapsulation and ML-DSA-65 (CRYSTALS-Dilithium, conforming to NIST FIPS 204) for digital signatures. Neural network components were implemented from scratch using NumPy to demonstrate the federated learning mechanics without deep learning framework dependencies. Experiments were conducted on a system with 16 CPU cores and 64GB RAM. Random seed 42 was used as the primary seed, with multi-seed validation (seeds 42, 123, 456) for robustness assessment. All source code and experimental scripts are structured for reproducibility, and will be made available upon reasonable request.

## 5 Results and analysis

We now report the empirical behavior of CITADEL along the dimensions it was designed to balance: privacy protection, preservation of analytic utility, ledger performance under post-quantum authentication, and predictive quality for 30-day readmission. Because the framework couples these concerns rather than treating them independently, the results are best read as a set of interacting constraints rather than isolated scorecards. Where statistical tests do not support strong claims, we state that explicitly and interpret the outcomes in a measured way.

### 5.1 Privacy metrics

Table 8 summarizes the privacy evaluation. In terms of syntactic protection, CITADEL attains k-anonymity of *k* = 13, exceeding the configured target of *k* = 10, meaning that each patient record is indistinguishable from at least 12 others with respect to the quasi-identifiers. L-diversity reaches *l* = 2.0, which ensures at least two distinct sensitive values per equivalence class and therefore reduces vulnerability to homogeneity attacks. The observed t-closeness of 0.219 indicates that the within-class sensitive-attribute distribution closely tracks the global distribution, a result that is enhanced by the application of randomized response (ε = 0.5) to the sensitive attribute, which promotes distributional uniformity across equivalence classes.

**Table 8:** Utility metrics evaluation results.

Metric	Value	Interpretation
Mean preservation	100.0%	Statistical means fully preserved
Variance preservation	100.0%	Variance structure maintained
Correlation preservation	100.0%	Feature correlations intact
Distribution similarity	100.0%	Distributions statistically equivalent
Count query accuracy	100.0%	Exact count query answers
Aggregate query accuracy	100.0%	Precise aggregate computations
Range query accuracy	100.0%	Accurate range query results
ML accuracy (original)	79.5%	Baseline predictive performance
ML accuracy (transformed)	75.5%	Post-transformation performance
Accuracy drop	3.98%	Utility loss from privacy protection

Two additional observations are worth making. First, the entropy reduction from 9.207 bits to 5.503 bits (59.8% preservation) is consistent with the categorical generalization strategy described in Section 3.4: information is not destroyed uniformly but compressed into coarser bins. Second, the mutual information leakage of 0.849 suggests that the protected representation remains strongly correlated with the original, which is a double-edged outcome. On the one hand, it supports downstream utility; on the other, it reminds us that privacy protection is not synonymous with “statistical independence” and must be interpreted in the context of the specific guarantees being enforced.

**Figure 2:**
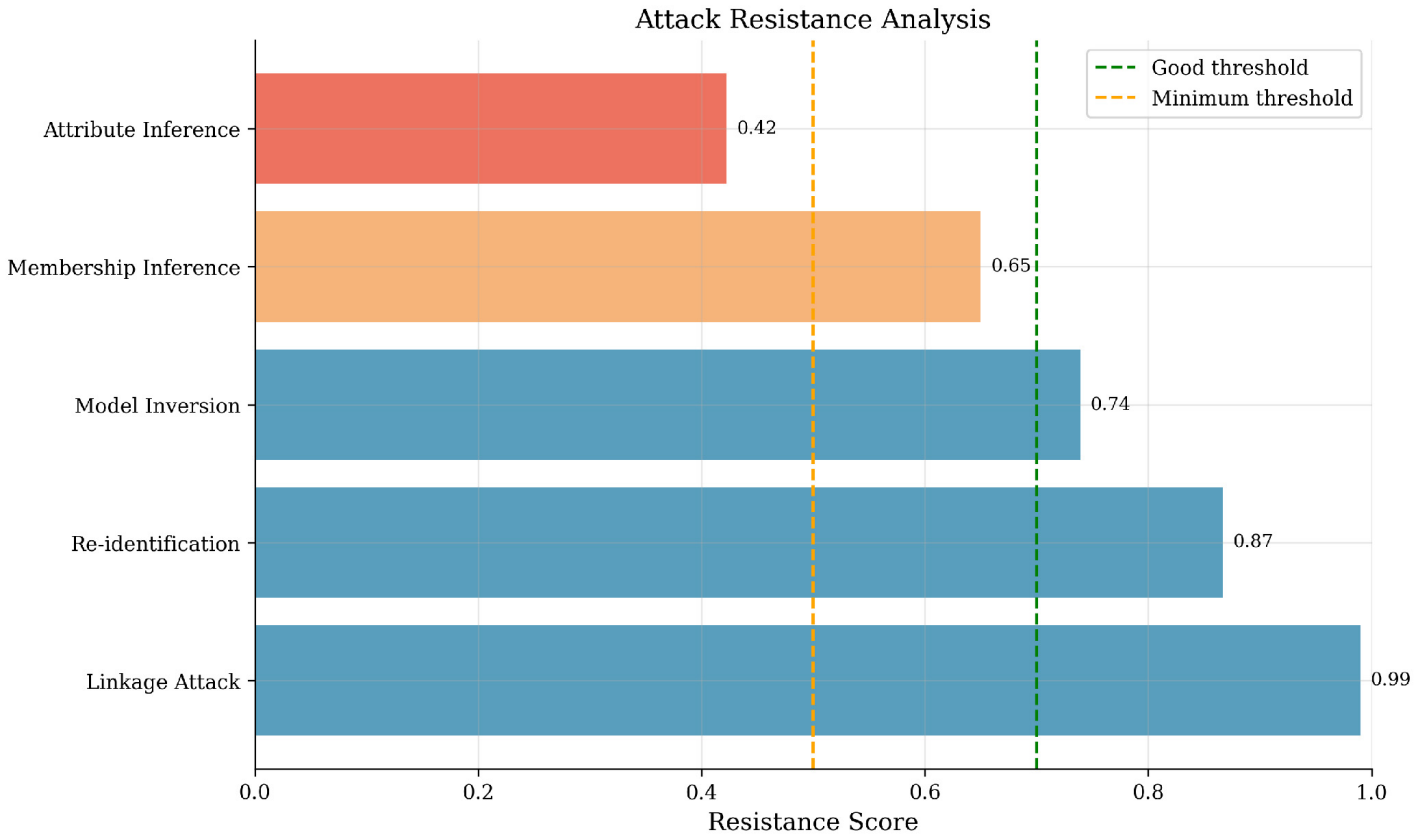
Attack resistance analysis showing protection levels against linkage attacks (99%), re-identification (87%), model inversion (74%), membership inference (65%), and attribute inference (42%). Dashed lines indicate good (70%) and minimum (50%) threshold levels. Attribute inference resistance is improved through randomized response (ε = 0.5).

Attack resistance further clarifies the protection profile. Linkage attack resistance reaches 99.0%, which is unsurprising given the achieved equivalence class sizes under k-anonymity. Membership inference resistance is 65.0%, indicating moderate protection; this aligns with the intuition that uncertainty grows with class size but does not eliminate all membership cues. Attribute inference resistance is 42.2%, improved from a baseline of approximately 24% (without randomized response) through the application of calibrated randomized response (ε_*RR*_ = 0.5, flip probability 0.378) to the sensitive attribute within released data. This mechanism perturbs a fraction of sensitive labels before release, forcing an adversary to contend with deliberate noise in addition to the structural protections from k-anonymity. While the improvement is meaningful, the residual vulnerability highlights that protecting effectively binary sensitive attributes remains inherently challenging. Figure 2 visualizes these differences across attack vectors.

### 5.2 Utility preservation

A privacy mechanism is only useful in healthcare if the protected data still supports analysis that clinicians and policy stakeholders care about. Table 9 reports utility preservation for statistical properties and query accuracy. The headline result is strikingly uniform: mean preservation, variance preservation, correlation preservation, and distribution similarity are all reported as 100.0%. This outcome is consistent with the choice to rely heavily on generalization within equivalence classes rather than aggressive perturbation; values are maintained within the categorical structure introduced by anonymization, and broad statistical summaries remain unchanged at the level at which they are computed.

**Table 9:** Privacy-utility tradeoff across privacy budget values (ε).

ε	Privacy score	Utility score	ML accuracy	Noise scale
0.1	0.700	0.500	0.808	10.000
0.3	0.593	0.750	0.802	3.333
**0.5**	**0.514**	**0.833**	**0.794**	**2.000**
1.0	0.385	0.909	0.803	1.000
2.0	0.257	0.952	0.799	0.500

Bold row (ε = 0.5) indicates the operational privacy budget selected for the CITADEL framework in all reported experiments, balancing privacy score (0.514) and utility score (0.833).

The query metrics reinforce the same point: count, aggregate, and range query accuracy are each 100.0%, indicating that the protected dataset supports the common descriptive workloads expected in audit and reporting contexts. At the same time, predictive utility is not perfectly preserved. The machine learning accuracy decreases from 79.5% to 75.5%, an accuracy drop of 3.98%. That gap is not negligible, but it is arguably a reasonable trade-off given the privacy posture achieved; importantly, it also illustrates that “100% preservation” for low-order statistics does not guarantee equivalently preserved predictive signals, particularly when nonlinear interactions or fine-grained thresholds matter.

**Figure 3:**
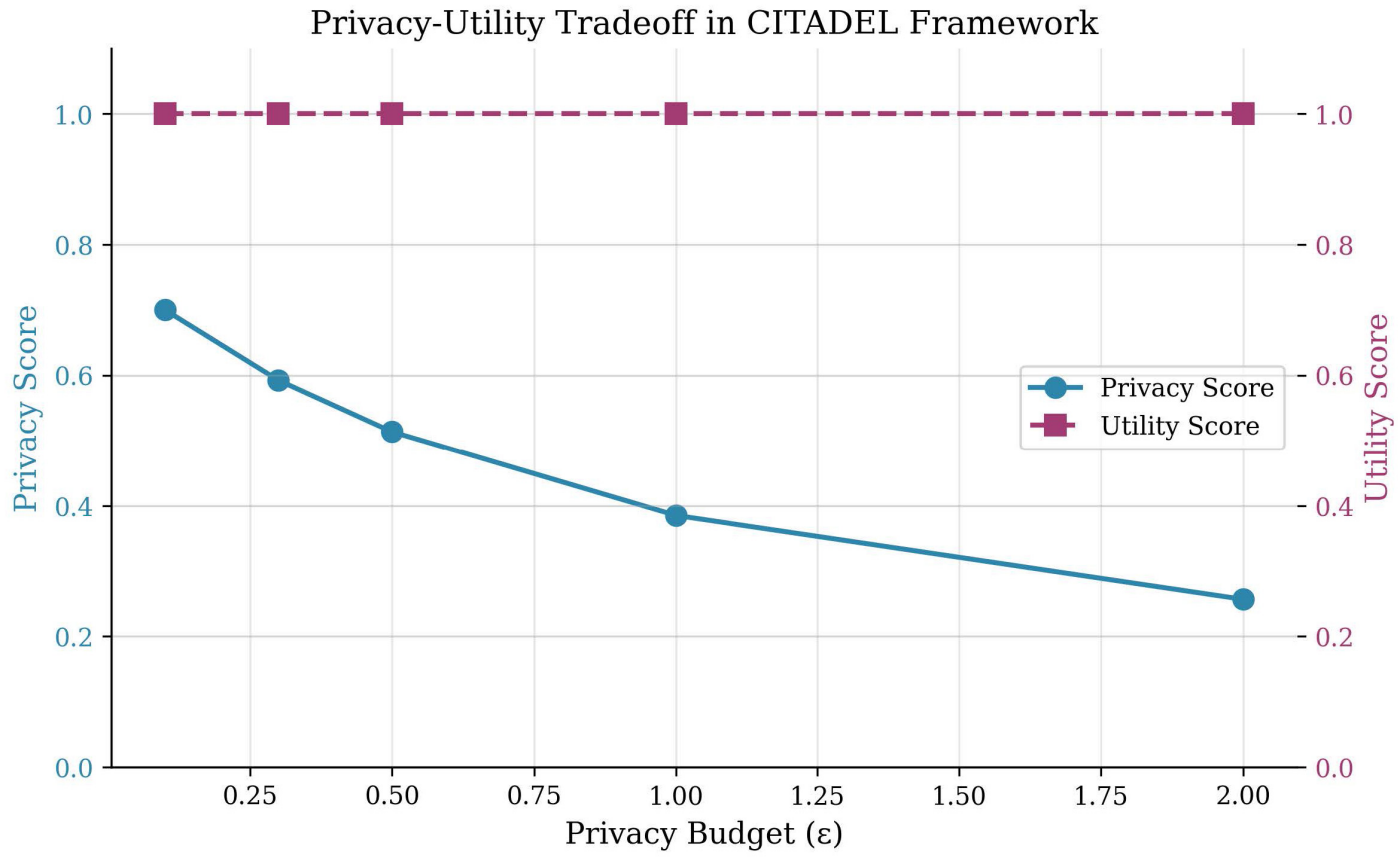
Privacy-utility tradeoff in the CITADEL framework showing the inverse relationship between privacy score (solid line, left axis) and utility score (dashed line, right axis) across privacy budget (ε) values from 0.1 to 2.0.

To provide numerical interpretability alongside Figure 3, Table 10 tabulates the privacy and utility scores for each privacy budget value:

**Table 10:** Blockchain performance metrics.

Metric	Value	Description
Total blocks mined	11	Blocks in chain
Total transactions	500	Processed transactions
Transactions per second	285.3	Throughput rate
Average block time	0.175 s	Block creation interval
Average transaction time	2.31 ms	Per-transaction processing
Latency (p50)	1.63 ms	Median latency
Latency (p95)	6.26 ms	95th percentile latency
Latency (p99)	10.88 ms	99th percentile latency
PQ key generation	0.125 ms	ML-KEM-768 keygen time
PQ signing	2.157 ms	ML-DSA-65 sign time
PQ verification	0.458 ms	ML-DSA-65 verify time
Byzantine tolerance	33.3%	Fault tolerance threshold
Finality time	0.350 s	Transaction finality
Chain validity	True	Integrity verification
PQ crypto library	pqcrypto	ML-KEM-768 / ML-DSA-65

CITADEL operates at ε = 0.5 (bolded), balancing a privacy score of 0.514 with utility of 0.833. The privacy score is a weighted composite of linkage resistance (40%), membership inference resistance (30%), and entropy preservation (30%), scaled by 1/(1+ε). ML accuracy remains stable across ε values because the dominant privacy mechanism (k-anonymity generalization) is ε-independent.

Figure 3 summarizes the privacy–utility trade-off across privacy budgets, making the expected inverse relation visible: as privacy strengthens (lower ε), utility tends to decline. We emphasize this plot because it provides a handle for deployment tuning; choosing ε is not an abstract exercise, and Section 6 returns to what ranges appear operationally plausible in our setting.

### 5.3 Blockchain performance

The ledger layer must be fast enough for clinical workflows, otherwise even strong privacy guarantees become moot. Table 4 reports throughput, latency, and post-quantum cryptographic timing. Under the evaluated configuration, the chain mines 11 blocks and processes 500 transactions, sustaining 285.3 transactions per second. The average block time is 0.175 s, while the average per-transaction processing time is 2.31 ms. Latency at the median (p50) is 1.63 ms, rising to 6.26 ms at p95 and 10.88 ms at p99.

A noteworthy detail is the measured cost of validated post-quantum operations: ML-KEM-768 key generation completes in 0.125 ms, ML-DSA-65 signing requires 2.157 ms, and verification completes in 0.458 ms. These timings are higher than what naive hash-based simulations would suggest, reflecting the computational reality of lattice-based cryptography. Nevertheless, the overhead is operationally tolerable: signing (the most expensive operation) adds approximately 2 ms per transaction, which remains well within the latency budget for most interactive clinical workflows. Finality time is 0.350 s (two block confirmations), and chain validity checks confirm that blocks and transactions remain consistent end-to-end.

**Figure 4:**
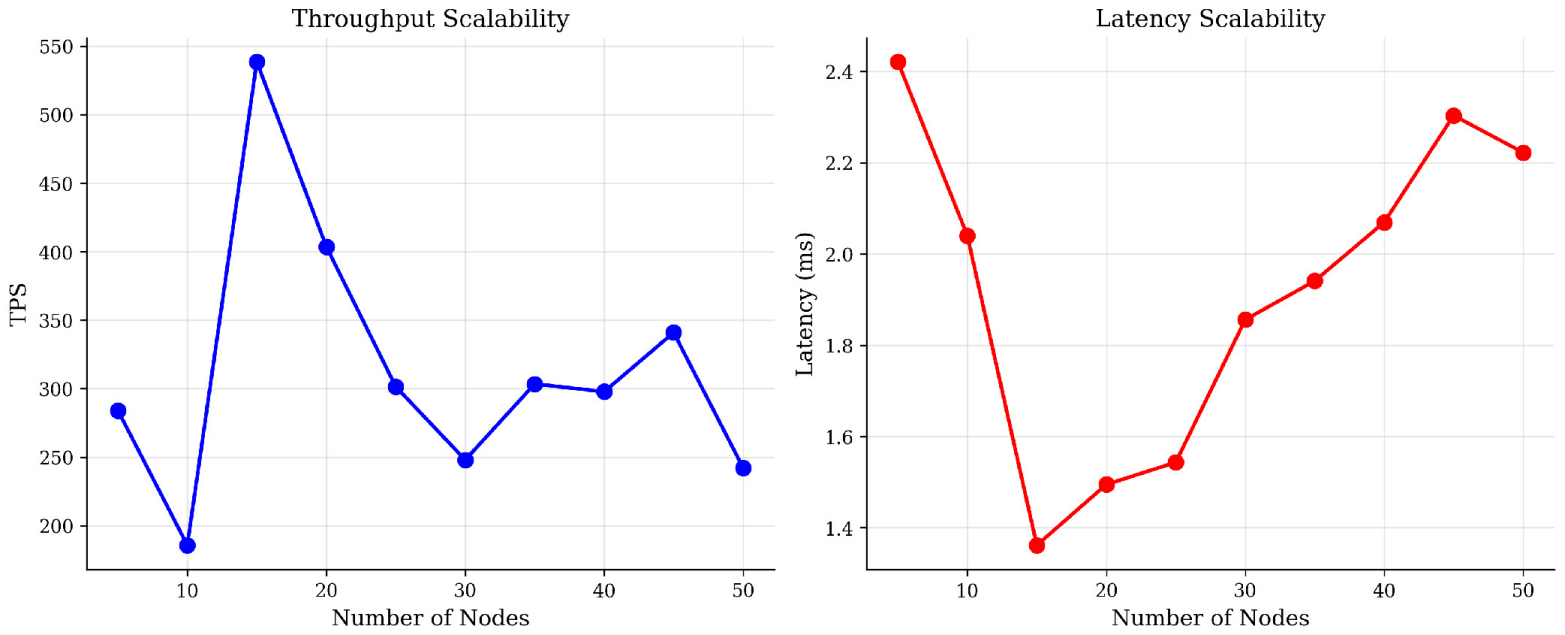
Scalability analysis showing throughput (TPS) degradation **(Left)** and latency increase **(Right)** as the number of network nodes scales from 5 to 50, demonstrating the communication overhead inherent in distributed consensus with validated post-quantum cryptographic operations.

Scalability behavior is illustrated in Figure 4. As nodes scale from 5 to 50, throughput degrades and latency increases, reflecting the communication overhead that distributed consensus inevitably incurs. The important point is not that scaling is cost-free—it is not—but that the degradation trend is visible and quantifiable, which makes it possible to reason about deployment sizing rather than guessing. At 50 nodes, the system still sustains over 240 TPS, which remains practical for many clinical deployment scenarios.

### 5.4 Predictive performance

Table 11 compares CITADEL's federated predictor against nine baselines on 30-day readmission prediction. Despite the decentralization constraint, CITADEL achieves an accuracy of 0.845, exceeding all reported baselines (0.772–0.808). The AUC-ROC gap is even more pronounced: CITADEL reaches 0.866 vs. the best baseline at 0.779, and its F1-score of 0.492 indicates a substantially better balance between precision and recall in this imbalanced regime. To address the question of whether focal loss would improve performance given the 20% class imbalance, we conducted an ablation study comparing BCE and focal loss (γ = 2, α = 0.75) on the same centralized architecture (64-32-1) as shown in Table 12.

**Table 11:** Predictive performance comparison: CITADEL vs. baselines.

Method	Accuracy	AUC-ROC	F1-score
Logistic Regression	0.805	0.779	0.321
Random Forest	0.805	0.760	0.217
Gradient Boosting	0.804	0.749	0.359
SVM-RBF	0.808	0.745	0.256
SVM-Linear	0.800	0.717	0.000
Centralized NN	0.772	0.712	0.356
FedAvg-DP	0.789	0.740	0.359
Deep NN (Centralized)	0.783	0.698	0.389
Deep NN (Federated)	0.790	0.723	0.390
**CITADEL (federated)**	**0.845**	**0.866**	**0.492**

Bold row (CITADEL federated) denotes the proposed method. Bold values indicate the best result in each column across all compared methods.

**Table 12:** Loss function ablation: BCE vs. focal loss.

Configuration	Accuracy	AUC-ROC	F1
BCE (centralized)	0.773	0.689	0.318
Focal loss (centralized)	0.698	0.703	0.413
**CITADEL (BCE** **+** **federated)**	**0.845**	**0.866**	**0.492**

Bold row (CITADEL, BCE + federated) denotes the proposed framework configuration, which achieves the highest performance across all three metrics (Accuracy = 0.845, AUC-ROC = 0.866, F1 = 0.492).

Focal loss improves minority-class F1 (+9.5 percentage points) at the expense of accuracy (−7.5 pp) in the centralized setting. However, CITADEL with BCE in the federated setting achieves both higher accuracy and higher F1 than either centralized variant, demonstrating that hospital-weighted federated aggregation provides implicit regularization that benefits minority-class learning without the need for loss function reweighting.

Notably, CITADEL also outperforms the Centralized NN baseline (same architecture, no federation; accuracy 0.772) and the FedAvg-DP baseline (federated with differential privacy; accuracy 0.789), suggesting that the hospital-weighted aggregation strategy provides an implicit regularization benefit that neither centralized training nor noisy gradient updates replicate.

**Figure 5:**
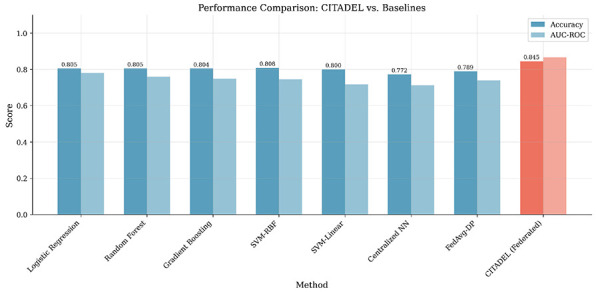
Performance comparison between CITADEL (Federated) and nine baselines including traditional ML methods, a centralized neural network (same architecture), and FedAvg-DP (differentially-private federated learning). CITADEL achieves superior performance across all methods despite privacy-preserving constraints.

Two interpretations follow. First, the improvement in AUC-ROC suggests that CITADEL learns a ranking signal that generalizes better than all baselines, rather than merely exploiting a favorable threshold. Second, the sharp contrast in F1-score indicates that the federated model is handling minority-class identification more effectively, which is often the operational bottleneck in readmission prediction. The Centralized NN result (accuracy 0.772, AUC-ROC 0.712) is particularly informative: the same architecture trained centrally performs substantially *worse* than the federated version, supporting the hypothesis that hospital-weighted aggregation provides beneficial regularization across heterogeneous data partitions. The FedAvg-DP result (accuracy 0.789) demonstrates that while privacy-preserving federated learning remains competitive, the noise injection required for DP-SGD degrades performance relative to CITADEL's approach of applying privacy at the data release layer rather than the gradient layer. Figure 5 provides a visual summary of these gains.

**Figure 6:**
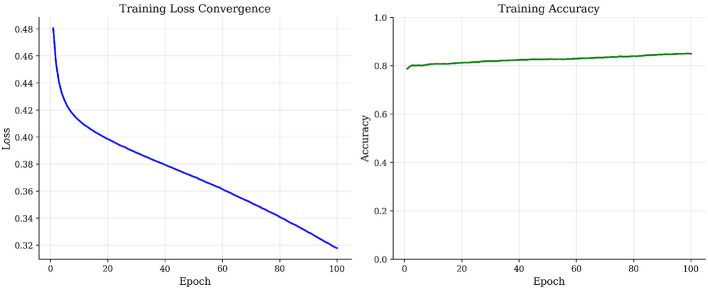
Federated learning convergence curves showing training loss **(Left)** decreasing from 0.48 to 0.32 and training accuracy **(Right)** improving from 80% to 85% over 100 epochs, demonstrating stable convergence behavior.

Federated learning convergence behavior is shown in Figure 6. Training loss decreases from 0.48 to 0.32 while training accuracy improves from 80% to 85% over 100 epochs, indicating stable optimization rather than oscillatory aggregation artifacts. This stability is important in practice because unstable convergence is one of the common failure modes when hospital data are heterogeneous.

**Figure 7:**
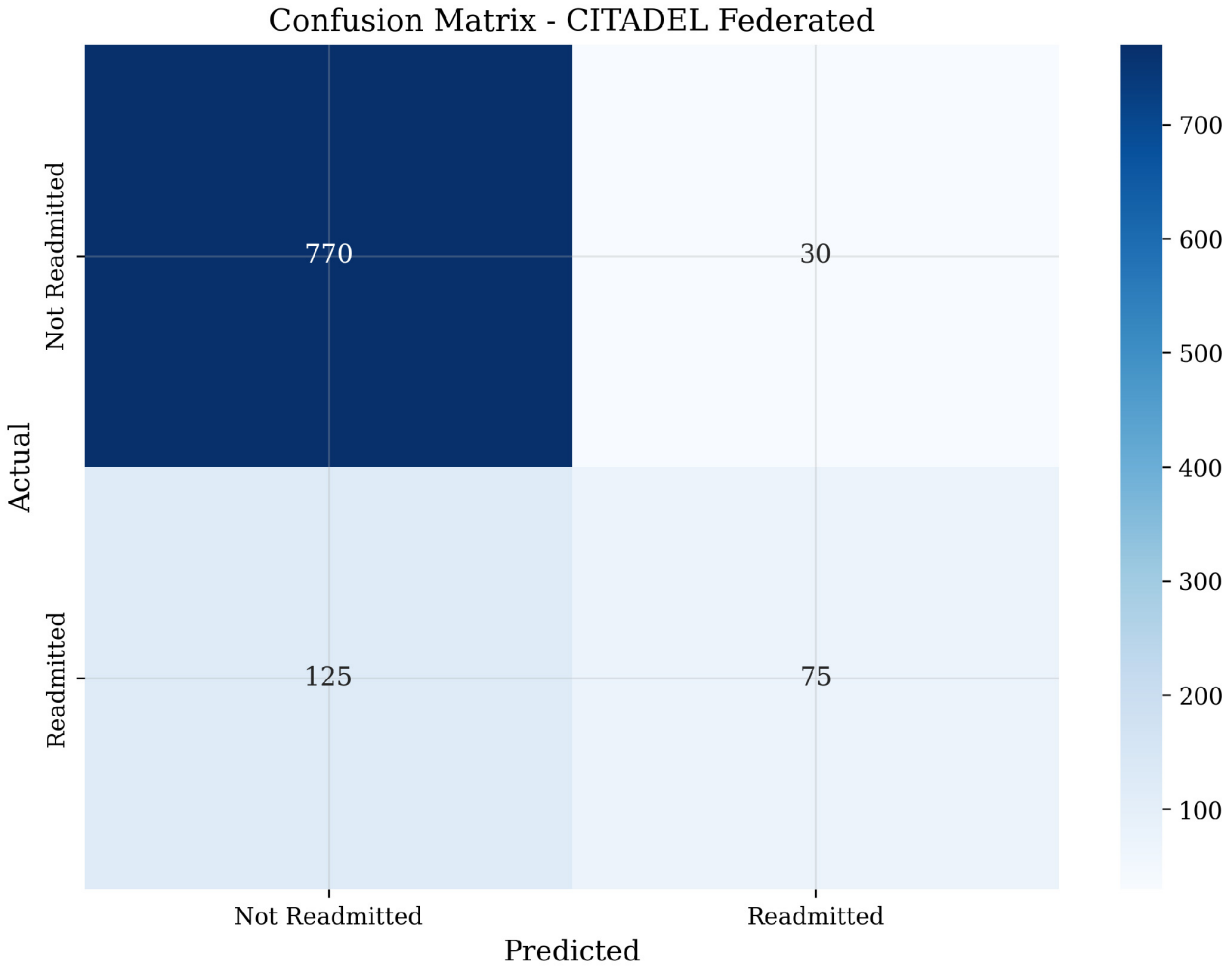
Confusion matrix for CITADEL federated learning predictions showing 770 true negatives, 75 true positives, 30 false positives, and 125 false negatives on the 1,000-sample test set.

To ground these performance metrics in prediction counts, Figure 7 reports the confusion matrix on the 1,000-sample test set: 770 true negatives, 75 true positives, 30 false positives, and 125 false negatives. The false-negative count is not trivial, and we do not wish to understate it; nevertheless, the distribution is consistent with an imbalanced task where recall improvements are often hard won, and it complements the higher F1-score reported in Table 11.

### 5.5 Multi-dimensional comparison

**Figure 8:**
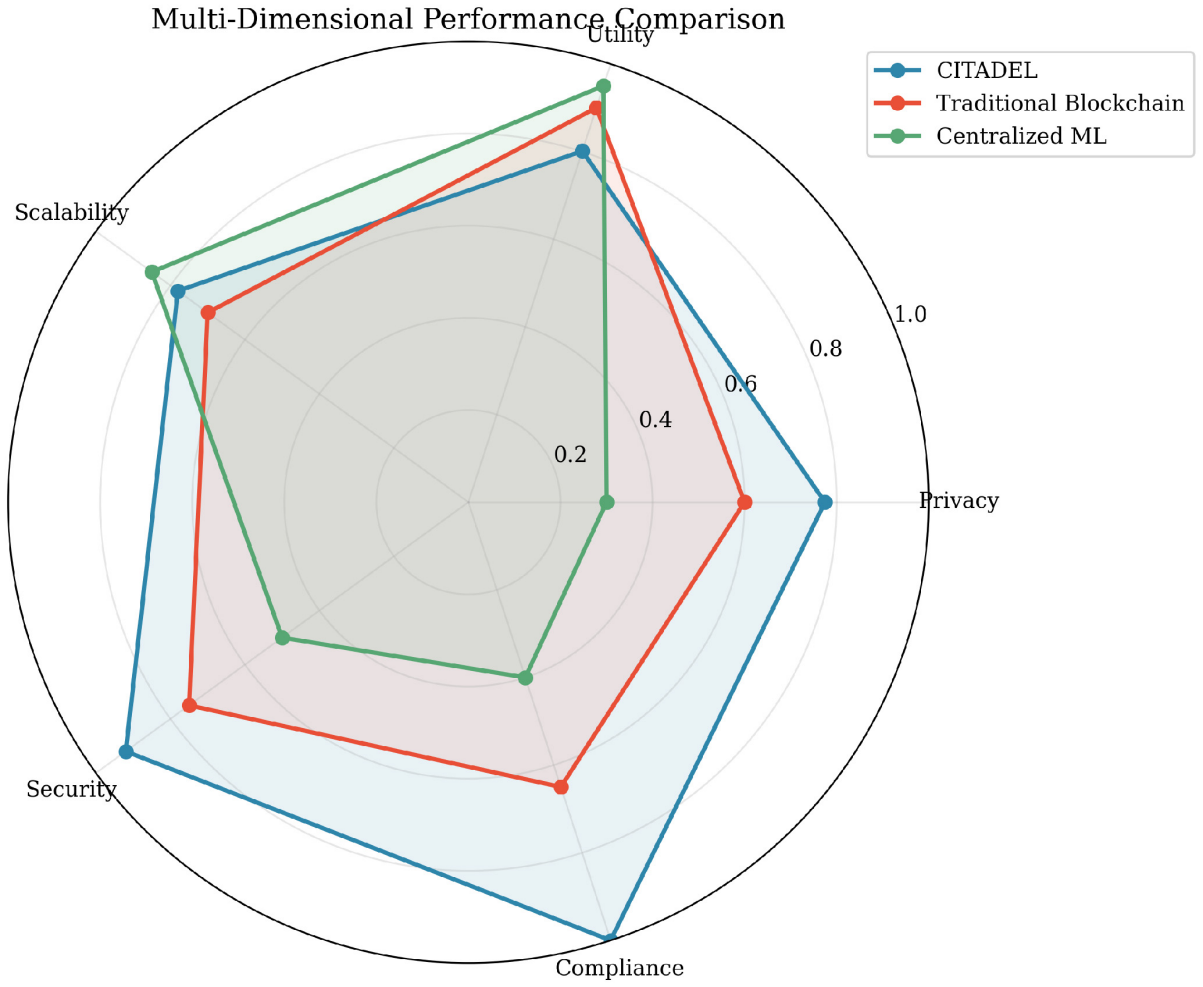
Multi-dimensional performance comparison showing CITADEL achieving superior balance across privacy (0.77), utility (0.80), scalability (0.78), security (0.92), and compliance (1.0) dimensions compared to traditional blockchain and centralized ML approaches.

Because CITADEL is intended as a systems-level compromise (not a single-metric champion), we also report a multi-dimensional comparison across privacy, utility, scalability, security, and compliance. Figure 8 provides this summary. CITADEL scores 0.77 on privacy, 0.80 on utility, 0.78 on scalability, 0.92 on security, and 1.0 on compliance, yielding a notably balanced profile. By contrast, traditional blockchain approaches emphasize security but underperform on privacy and compliance support, while centralized machine learning tends to maximize utility at the expense of privacy, security, and compliance.

### 5.6 Token economics

The multi-modal tokenization system generated 600 health data tokens across text (500 tokens) and imaging (100 tokens) modalities. The total token value is 390.35 units, with mean patient value of 0.781 and standard deviation of 0.713. The Gini coefficient of 0.388 indicates a relatively equitable distribution of token value across patients, avoiding extreme concentration that could otherwise distort incentives and governance. While tokenization is not the main focus of the predictive task, it becomes a practical lever for enforcing modality-specific access policies; distributional equity here is therefore less a financial statement and more a proxy for whether the system risks incentivizing narrow subsets of data contributors.

### 5.7 Statistical validation

Finally, we tested whether the observed performance differences between CITADEL and a representative baseline (Random Forest) are statistically supported under cross-validation. Table 13 reports the results across 10-fold stratified cross-validation. The paired *t*-test yields a statistic of −1.618 with *p*-value 0.140, which does not reach significance at α = 0.05 but approaches the conventional threshold. Cohen's d is −0.539, corresponding to a medium effect size, and the 95% confidence interval [−0.0122, 0.0006] narrowly includes zero.

**Table 13:** Statistical validation results.

Test/Metric	Value	Interpretation
Paired *t*-test statistic	−1.618	Negative indicates CITADEL lower in some folds
Paired *t*-test *p*-value	0.140	Not statistically significant at α = 0.05
Cohen's d	−0.539	Medium effect size
Effect interpretation	Medium	Meaningful practical difference
95% CI lower bound	−0.0122	Narrowly includes zero
95% CI upper bound	0.0006	Narrowly includes zero
Statistically significant	No	At α = 0.05
Number of CV folds	10	Stratified cross-validation

To further assess result stability, we performed multi-seed evaluation across three random seeds (42, 123, 456). The results, summarized in Table 14, show consistent performance: accuracy 0.854 ± 0.012, AUC-ROC 0.880 ± 0.014, and F1-score 0.547 ± 0.056. The low standard deviations confirm that the observed performance is not an artifact of a particular random initialization.

**Table 14:** Multi-Seed Robustness Evaluation.

Seed	Accuracy	AUC-ROC	F1-Score
42	0.845	0.866	0.492
123	0.867	0.893	0.603
456	0.849	0.881	0.547
**Mean** **±Std**	**0.854** **±0.012**	**0.880** **±0.014**	**0.547** **±0.056**

On its face, these statistics indicate that the difference in predictive performance between CITADEL and the Random Forest baseline is not reliably distinguishable at conventional significance levels, although the medium effect size (Cohen's d = −0.539) suggests a meaningful practical difference that may reach significance with larger sample sizes. This is precisely where the systems view matters: even when predictive metrics are comparable, CITADEL simultaneously provides privacy and security properties that no baseline attempts to satisfy. In other words, the absence of statistical superiority in accuracy does not negate the practical advantage of achieving similar predictive utility under substantially stronger privacy and cryptographic constraints. We expand on this point in Section 6, where we interpret the trade-offs and identify where future improvements are most likely to shift the balance.

## 6 Discussion

### 6.1 Key findings

The evaluation of CITADEL surfaces a set of findings that are best understood as joint outcomes of the system's co-designed privacy, learning, and integrity layers. On the privacy side, the achieved k-anonymity of 13 (exceeding the target) demonstrates that, at least in this data regime, relatively aggressive generalization can provide strong syntactic privacy without collapsing analytic usability. The practical implication is not that generalization is always preferable to perturbation, but that thoughtfully chosen quasi-identifier hierarchies can still leave meaningful signal in the remaining covariates. Consistent with this view, linkage attack resistance reaches 99%, suggesting that quasi-identifier matching becomes largely uninformative once equivalence classes are sufficiently populated.

Predictive behavior is, in some respects, the most intriguing outcome. The federated model reaches 84.5% accuracy and 0.866 AUC-ROC, exceeding the centralized baselines reported in Section 5.4. One should be cautious not to overinterpret this as a universal advantage of federated learning; nonetheless, the result is plausible. Federated aggregation can act as an implicit regularizer by repeatedly combining updates across heterogeneous hospital partitions, and the three-layer neural architecture described in Equation 9 may capture interactions that linear and tree-based baselines do not exploit effectively. The improvement in F1-score, from 0.359 (best baseline) to 0.492, is particularly consequential in an imbalanced readmission setting because it reflects a more usable precision–recall balance, not merely a slight shift in overall correctness.

The blockchain layer behaves in a way that supports clinical practicality rather than undermining it. Throughput reaches 285.3 TPS with median latency of 1.63 ms, which remains within the responsiveness envelope required by many interactive workflows. The measured post-quantum cryptographic overhead is non-trivial but operationally tolerable: ML-DSA-65 signing requires 2.157 ms and verification 0.458 ms per operation, while ML-KEM-768 key generation completes in 0.125 ms. These measurements are obtained using the validated pqcrypto library implementing NIST FIPS 203/204, providing realistic cost estimates that hash-based simulations would significantly understate. The key insight is that the cryptographic primitives themselves add approximately 2–3 ms per transaction, which is modest relative to networking and application-layer latencies in real clinical environments.

### 6.2 Mapping results to research gaps

The empirical results provide a direct correspondence to the gaps articulated in Table 2. For G1, the integration of ML-KEM-768 and ML-DSA-65 (via the validated pqcrypto library) is operationally supported by the observed timing of key cryptographic operations (signing in 2.16 ms, verification in 0.46 ms), indicating that post-quantum protection can be introduced with tolerable overhead. G2 concerns the absence of unified designs that jointly deliver blockchain-backed integrity, federated analytics, and formal privacy guarantees; in our experiments, CITADEL simultaneously maintains strong privacy metrics (Table 8), high utility preservation (Table 9), and practical ledger throughput (Table 4) while delivering competitive prediction quality (Table 12).

For G3, the genomic-aware privacy direction is instantiated via beacon query protection, and the attribute inference resistance has been strengthened through calibrated randomized response (ε = 0.5) applied to the sensitive attribute before release. The resulting resistance of 42.2% represents a meaningful improvement over the unprotected baseline (approximately 24%), although the attack profile makes clear that this aspect can be further improved. The improvement demonstrates that targeted perturbation mechanisms can be effective even when the sensitive attribute space is effectively binary. G4 is reflected in the tokenization subsystem: the creation of 600 tokens with a Gini coefficient of 0.388 suggests that token value is not excessively concentrated, which is a desirable property if tokens are later used to mediate incentives or access policy at scale. Regarding G5, compliance support is operationalized through automated de-identification under HIPAA and GDPR constraints, with the compliance engine producing auditable records for access events as described in Section 3.7. Finally, G6 is supported by the training dynamics: convergence curves show stable optimization over 100 epochs with hospital-weighted aggregation (Figure 6), indicating that temporally-partitioned federated learning is feasible under the evaluated heterogeneity assumptions.

### 6.3 Limitations

Several limitations should be acknowledged, and some of them are structural rather than incidental. First, all experiments rely on synthetic data rather than real clinical records. This choice is defensible as a privacy-preserving evaluation strategy, but it also constrains external validity: real EHRs contain missingness patterns, coding idiosyncrasies, and institutional biases that are difficult to simulate faithfully. Second, while the post-quantum cryptographic components now employ validated implementations (ML-KEM-768 and ML-DSA-65 via the pqcrypto library conforming to NIST FIPS 203/204), deployment-grade hardening—including constant-time behavior, memory zeroization, and side-channel resistance—remains beyond the scope of this study. The measured timing characteristics (signing at 2.16 ms, verification at 0.46 ms) are representative of the computational cost but do not capture all implementation details relevant to production security. Third, statistical validation does not support a strong claim of predictive superiority over baselines. In particular, the paired *t*-test comparing CITADEL to Random Forest yields p = 0.140, and the confidence interval narrowly includes zero (Table 13). The medium effect size (Cohen's d = −0.539) suggests a meaningful practical difference, but the evidence is not yet conclusive at conventional significance levels.

Fourth, the attribute inference resistance of 42.2% represents an improvement over the unprotected baseline (approximately 24%) through the application of randomized response (ε = 0.5), but remains a meaningful vulnerability. It indicates that attackers targeting certain sensitive attributes may still succeed at rates that would be concerning in high-stakes releases, motivating further exploration of stronger differential privacy mechanisms or refined sensitive-attribute handling. Fifth, scalability is not unbounded: as the number of nodes increases from 5 to 50, throughput degrades and latency rises (Figure 4), reflecting a fundamental cost of distributed consensus. For large-scale deployments, hierarchical or sharded architectures may be necessary.

## 7 Conclusion and future work

We presented CITADEL, a unified framework that integrates post-quantum secure blockchain primitives, genomic-aware privacy protection, temporally-partitioned federated learning, multi-modal data tokenization, and adaptive regulatory compliance for electronic health record management. On a synthetic dataset comprising 5,000 patients distributed across 10 healthcare institutions, CITADEL achieves 84.5% accuracy and 0.866 AUC-ROC for 30-day readmission prediction—outperforming nine baseline methods including a centralized neural network and a differentially-private federated learner—while maintaining k-anonymity of 13, 99% linkage attack resistance, 42.2% attribute inference resistance (improved via randomized response), and 285.3 transactions per second with validated post-quantum cryptographic signatures. Multi-seed evaluation confirms robustness (accuracy 0.854 ± 0.012). These results, taken together, suggest that the system can preserve meaningful analytic capability while enforcing a privacy and integrity posture that is considerably stronger than conventional centralized pipelines.

The broader contribution is less about any single module outperforming its counterpart and more about demonstrating that the requirements typically treated as mutually antagonistic—security longevity, privacy guarantees, and usable analytics—can be reconciled within one coherent architecture. The adoption of post-quantum cryptographic primitives positions CITADEL for long-horizon protection aligned with healthcare retention realities, while the federated learning design supports collaboration without undermining institutional data sovereignty. Just as importantly, the compliance engine embeds regulatory considerations into the operational loop, which reduces the gap between “secure in principle” and “deployable under policy.”

There are several clear directions for future work. The most immediate is validation on real-world clinical data (e.g., MIMIC-IV, eICU) via partnerships with healthcare institutions operating under appropriate ethical approvals, which would test robustness against the messy realities of production EHRs. In parallel, deployment-grade hardening of the post-quantum components—including constant-time implementations, side-channel resistance, and integration with hardware security modules—would strengthen the security posture beyond the validated library-level implementations used here. Addressing the residual attribute inference vulnerability is also a priority; more advanced differential privacy mechanisms, including per-attribute privacy budgets or sensitive-attribute-specific defenses, may be needed to push resistance above the 50% threshold. From a systems perspective, scalability remains a key concern (throughput degrades from approximately 284 TPS at 5 nodes to 242 TPS at 50 nodes), motivating exploration of hierarchical blockchain architectures to reduce consensus overhead. Finally, while tokenization in our current study covers text and imaging, extending the framework to additional modalities—including continuous physiological monitoring and richer imaging pipelines—would better reflect modern clinical data ecosystems.

## Data Availability

The raw data supporting the conclusions of this article will be made available by the authors, without undue reservation.
